# Integrating Mechanical Diagnosis and Therapy Within Multidisciplinary Biopsychosocial Care for Chronic Musculoskeletal Pain

**DOI:** 10.26502/josm.511500253

**Published:** 2026-03-05

**Authors:** Manas Aavula, David Parvizi, Sugeeth Kandikattu, Devendra K Agrawal

**Affiliations:** Department of Translational Research, College of Osteopathic Medicine of the Pacific, Western University of Health Sciences, Pomona, California 91766, USA

**Keywords:** Biopsychosocial model, Central sensitization, Chronic musculoskeletal pain, Fibromyalgia, Mechanical Diagnosis and Therapy (MDT), McKenzie Method, Multidisciplinary Rehabilitation, Osteoarthritis

## Abstract

Chronic musculoskeletal conditions such as osteoarthritis and fibromyalgia make up a major portion of the disability population worldwide. An interplay among biological, psychological, and social factors influences the symptoms of these disorders. Treating these disorders requires a multidisciplinary approach, as a unimodal approach fails to produce long-term functional improvement. This review studies the role of biopsychosocial care in chronic pain management with an emphasis on the indications and limitations of the McKenzie Method of Mechanical Diagnosis and Therapy (MDT). MDT was compared with the effectiveness of physical therapy, occupational therapy, medication management, and psychological interventions, and its effectiveness was also examined when integrated into a multidisciplinary rehabilitation model. MDT has a limited role in centrally mediated conditions such as fibromyalgia, but it is superior in situations controlled by mechanism. MDT increases self-management, lowers disability, and reduces one’s dependence on passive therapies. MDT is best seen as a mechanism-specific, patient-centered intervention integrated into a biopsychosocial framework. Ideal long-term outcomes in chronic musculoskeletal pain involve the integration of progressive exercise, occupational therapy, psychological intervention, targeted mechanical therapy, and medication management.

## 1. Introduction

Movements are affected by one’s joints and the activities they can perform, shaping their quality of life. Joint diseases are separated into two categories: degenerative and inflammatory. Degenerative diseases, such as osteoarthritis, are described as “wear and tear” disorders associated with aging, prior injury, and other factors that result in cartilage degradation, triggering inflammation and driving much of the clinical presentation of osteoarthritis. Physiologically, inflammatory joint diseases result from autoimmune activity where the immune system mistakenly targets healthy tissue. Musculoskeletal conditions such as osteoarthritis and inflammatory arthritis are a significant cause of disability that heavily affects the healthcare system. 40% of adults aged 70 and older have osteoarthritis, and 25% of these adults cannot perform daily activities. Many of these conditions increase with age, obesity, and low physical exercise [1–6].

Osteoarthritis affects approximately 3.6% of the global population. It is caused by pro-inflammatory markers and proteases that destroy articular cartilage, leading to erosions that extend into the bone and much of the joint surface [7–10]. The collagen matrix is broken down, leading to chondrocyte proliferation and the formation of outgrowths that ossify into osteophytes. Patients present with joint pain, stiffness, restricted movement, weakness, bony swelling, joint deformity, and poor balance. Patients complain of pain during activity, but it decreases with rest. Osteoarthritis tends to affect the hips, knees, proximal and distal interphalangeal joints, first carpometacarpal joints, and the lower cervical and lumbar spine. Clinical exams will find tenderness at the joint lines, Heberden’s nodes, Bouchard’s nodes, effusions, crepitus, and bony enlargements. Osteoarthritis is a clinical diagnosis with complete blood count, erythrocyte sedimentation rate, rheumatoid factor, and antinuclear antibody tests normal. X-rays in patients with suspected osteoarthritis show joint space narrowing, marginal osteophytes, subchondral sclerosis, or cysts. Treatment involves pharmacological and nonpharmacological methods with the goals of lowering the pain and preventing functional loss. The prognosis for osteoarthritis depends on which joints are affected and the severity of symptoms, with joint replacement surgery the best treatment in specific scenarios [11].

Fibromyalgia is a chronic pain disorder caused by diffuse musculoskeletal pain along with fatigue, cognitive dysfunction, psychiatric disruptions, and various other somatic problems. It is present in 2% to 3% of individuals in the United States, with the highest prevalence among females aged 20 to 55. Patients with fibromyalgia experience altered pain perception and sensory processing due to changes in the central nervous system caused by infections, emotional trauma, and more. Although no single gene has been found to predispose patients to fibromyalgia, patients with a first-degree relative with fibromyalgia have a higher chance of developing the disease. Patients experience pain for more than 3 months along with other symptoms, including fatigue, sleep disturbances, and psychiatric symptoms. Imaging and laboratory test results are expected but needed to exclude other diagnoses that are like fibromyalgia symptoms. The treatment approach involves pharmacological methods, such as pregabalin and tricyclic antidepressants, as well as nonpharmacological methods, such as education, exercise, and psychotherapy [12].

**Figure 1:**
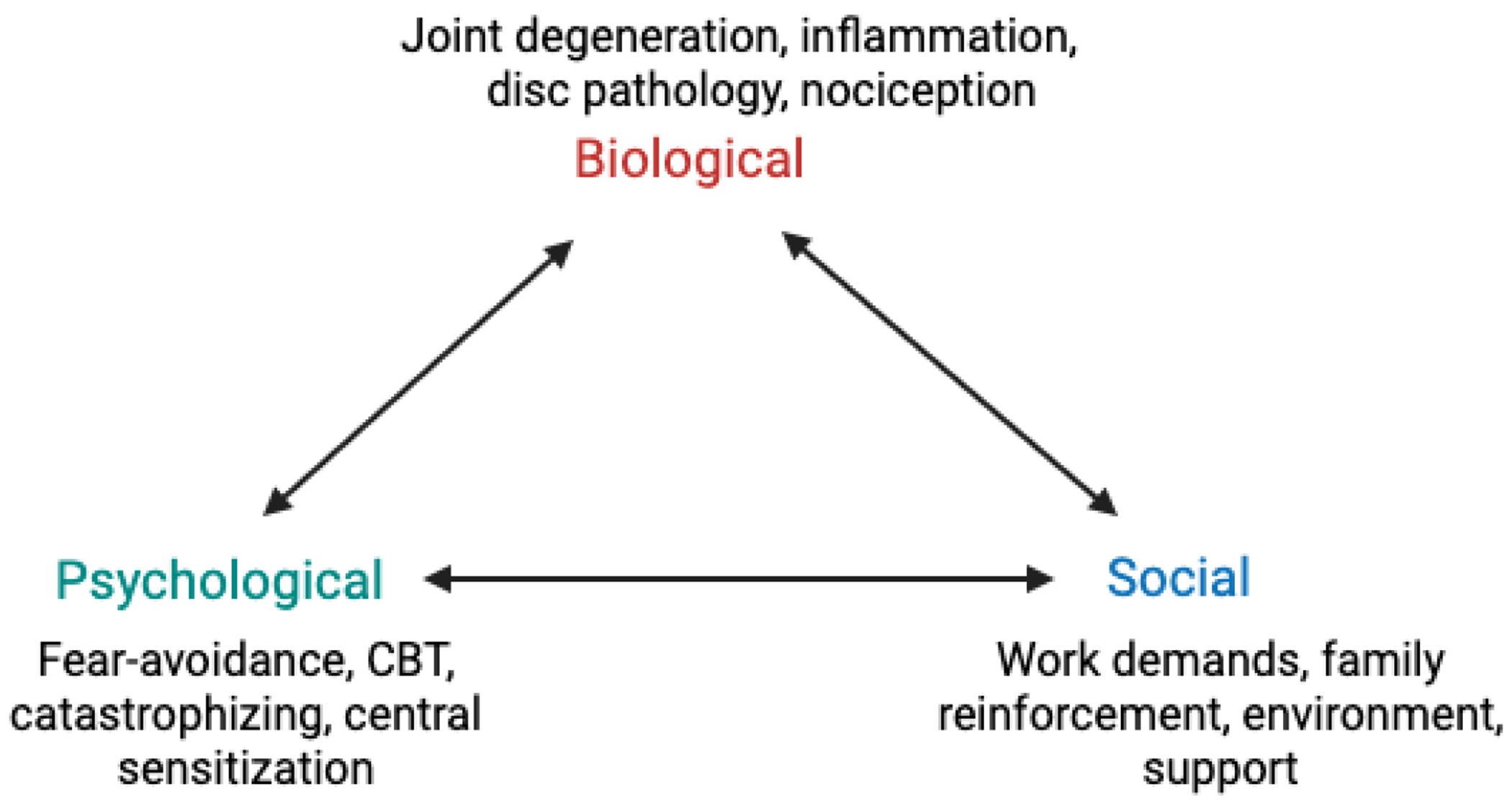
Biopsychosocial model of chronic musculoskeletal pain.

Chronic conditions such as fibromyalgia and osteoarthritis are disorders influenced by an interplay of biological, psychological, and social factors (Figure 1). As a result, modern management emphasizes a multidisciplinary model that integrates pharmacological methods, rehabilitation therapies, and psychological support to treat the symptoms, leading to better functional outcomes. The McKenzie Method of Mechanical Diagnosis and Therapy, a physical therapy approach, is used for patients with accompanying mechanical pain patterns. The purpose of this paper is to explore how multidisciplinary, biopsychosocial strategies are applied to treat musculoskeletal problems with a focus on the indications, limitations, and long-term outcomes of the McKenzie Method compared with other treatment modalities.

## 2. Physical Therapy

Physical therapy is a core component of chronic pain management. It involves various exercises, manual therapy such as massage, and treatments to reduce pain, strengthen weakened muscles, and improve their function. Exercises can be done by oneself or performed as passive movement done by the therapist. Interventions such as graded activity and individualized exercise programs address deconditioning and maladaptive movement patterns [13]. Exercise, such as aerobic and strength training, is recommended as part of fibromyalgia management, and when progressively increased over time, it can lead to significant benefits. Aerobic exercise was shown to reduce pain, fatigue, and depression. Strength training led to improvements in physical function and well-being. In contrast, mixed exercise training, which is aerobic and strength training, led to less pain and better physical function, despite the persistence of central sensitization [14]. Similarly, in patients with osteoarthritis, land-based therapeutic exercise that centered on strengthening, joint range of motion, and neuromuscular control led to improved pain and quality of life [15]. Manual therapy is included in rehabilitation programs and has shown to further improve pain, stiffness, and function in patients diagnosed with osteoarthritis and on a supervised exercise program, delaying the need for surgical intervention [16]. Response to physical therapy varies from patient to patient, underscoring the need for targeted interventions within broader rehabilitation programs.

## 3. Occupational Therapy

Occupational therapy helps patients develop skills to live independently and deal with physical, mental, or psychological limitations. Goals of this therapy include using medical aids such as walkers, performing movement exercises, and practicing daily skills for school, work, and everyday activities [17]. Therapists find new ways to conduct activities to adapt to physical and social environments, enhancing function and participation [18]. Occupational therapists educate patients with osteoarthritis on the principles of joint protection, minimizing fatigue, reducing joint pain and stress, and implementing ergonomic modifications. Emphasis is placed on the patient’s environment to implement specific interventions that strengthen the patient’s capabilities. For example, therapists may prescribe assistive devices to reduce joint stress. These devices can aid in the kitchen, lower stress on carpometacarpal joints to reduce gripping force, and more. These strategies are helpful for patients with chronic joint pain or work-related physical demands [19]. Beyond physical adjustments, occupational therapy addresses behavioral factors that contribute to disability and improves social participation. Patients had reduced feelings of helplessness, increased confidence in managing symptoms, re-engaged with family and work, and lower mental fatigue [20]. Occupational therapy, exercise-based rehabilitation, and patient education have improved functional independence and reduced pain [19].

## 4. Medication Management

Pharmacological therapy plays a supportive role in chronic pain management, helping to mitigate pain rather than definitively fixing the problem. Centrally acting medications such as serotonin-norepinephrine reuptake inhibitors (SNRIs), tricyclic antidepressants (TCAs), and gabapentinoids have played a major role in limiting pain and improving sleep in patients with fibromyalgia. These medications target the central mechanism underlying this condition [21,22]. On the other hand, frequently used medications for arthritis include nonsteroidal anti-inflammatory drugs (NSAIDs), corticosteroids, and disease-modifying antirheumatic drugs, which target peripheral inflammation and nociception [23].

Currently, pharmacological advancements to mitigate the symptoms and alter the structural progression have been a major goal. However, after numerous drug trials, no medication has been approved. At present, despite the advantages of current medications, they are not a sustainable solution for functional improvement due to the numerous side effects, which can be exacerbated with long-term use. Therefore, clinical guidelines highlight the use of both pharmacological and nonpharmacological methods to manage chronic pain. This highlights the role of rehabilitation and psychological interventions in fostering a beneficial long-term outlook [24].

## 5. Psychological Interventions

Psychological interventions are necessary to address pain beyond physical aspects. It is important to address the cognitive, emotional, and behavioral aspects of chronic pain. Cognitive behavioral therapy (CBT), mindfulness-based stress reduction, and pain coping skills have been proven to mitigate pain-related stress, catastrophizing, depressed mood, and healthcare-seeking actions. In one randomized clinical trial, mindfulness-based stress reduction stopped patients from overusing opioids to treat chronic pain, leading to improvements in chronic pain symptoms [25,26]. These psychological approaches alter abnormal pain beliefs and emotional reactions that amplify symptoms and limit functional movements.

Chronic pain is distributed across neural networks involved in sensory input, emotion, and cognition, which helps explain why pain can persist after tissue healing. Chronic pain is associated with plasticity in spinal pathways, amplifying pain perception and modifying sensory perception. Emotional processes such as fear-avoidance, catastrophizing, anxiety, and depression guide how pain is understood and responded to. Social context, such as reinforcement of pain behaviors or work and family responses shape long-term pain behavior and functional results. Biopsychosocial interventions address various aspects of pain, such as emotional regulation, maladaptive beliefs, behaviors, and neural processing, instead of just the mitigation of symptoms [27].

## 6. Implementing a Biopsychosocial Network

Collectively, physical therapy, occupational therapy, medication management, and psychological interventions highlight the need for a biopsychosocial approach to treat chronic pain (Figure 1). Physical, psychological, and social factors are treated together rather than separately. Rehabilitation programs composed of multidisciplinary biopsychosocial rehab have delivered superior outcomes in comparison to models that treat each component in isolation. Patients had higher odds of returning to work at 1 year than individuals treated solely physically [28]. This foundation provides the background for targeted physical therapy, such as the McKenzie Method of Mechanical Diagnosis and Therapy, to evaluate its role, indications, and constraints in treating chronic pain populations.

## 7. The McKenzie Method

### 7.1 Principles and Clinical Framework

The McKenzie Method of Mechanical Diagnosis and Therapy (MDT) is an assessment and treatment model for various musculoskeletal conditions that classifies patients into 3 mechanical subgroups: derangement, dysfunction, or postural syndrome. Derangement, the most observed type, is characterized by rapid changes in symptoms after repeated exercise in a direction preferred by the patient, leading to symptom improvement. These improvements may encompass centralization, an incident where symptoms down the lower extremity fade away distal to proximal towards the spine, a key clinical indication associated with favorable outcomes [29]. Dysfunction syndrome results from pain caused by mechanical deformation of soft tissue due to trauma, inflammation, or degeneration, leading to tissue contraction, scarring, or adaptive shortening, resulting in restricted movement and pain at the end of the range of motion. Postural syndrome results from mechanical deformation of soft tissue or vasculature under extended static loading, and symptoms transpire from prolonged positions such as sitting, standing, or lying. By evaluating symptom response to mechanical loading, MDT aims to identify movements that mitigate nociceptive input and increase functional tolerance [30].

Compared with passive treatment models, MDT emphasizes active patient participation, with patients instructed to complete an individualized program independently at home up to 10 times a day. This is different from weekly guided physical therapy sessions. The focus on independence and self-efficacy aligns with modern rehabilitation principles and is pertinent to patients with chronic pain, as long-term outcomes are related to patient participation and compliance [30].

### 7.2 Physiological Rationale

The physiology underlying MDT is rooted in the concept that intervertebral discs enable spinal motion through flexion, extension, side bending, and rotation. Degeneration of the annulus fibrosis and nucleus pulposus leads to axial back pain and radicular pain. Extension-based pain arises from the mechanical stress of facet joints, while posterior movement of the nucleus pulposus is related to flexion movements. Displacements of these structures lead to contortion of adjacent soft tissues. Patients who exhibit centralization of back pain are instructed to perform McKenzie exercises aligned with their directional preference. Patients with a tendency toward spinal extension will complete exercises that promote spinal extension [30].

Evidence proves that centralization is not only a clinical observation but also a sign of treatment responsiveness. In a study by Long et al. [31], patients underwent an MDT mechanical assessment and were placed into one of three groups. The first group received exercises that matched the patient’s directional preference; the second group received the opposite; and the third group received nondirectional, evidence-based care. Results showed that patients who performed exercises matching their directional preference were safer than those in other groups. This group improved significantly in every category of pain, disability, medication use, activity interference, and depression scores. Patients were more likely to report symptom resolution and return to work and recreational activities. The other two groups had exacerbation of symptoms, worsening of distal pain, and lower satisfaction rates with recovery. Centralization was not only a positive prognostic indicator but also a sign that guides treatment selection, as mismatched exercises worsened centralization [31]. MDT’s emphasis on self-management may alter central pain processing mechanisms due to lower reliance on passive care. Performing repeated movements may reduce maladaptive beliefs and fear-avoidant behaviors, two factors known to cause pain persistence and central sensitization. These principles place MDT as a patient-centered approach in the biopsychosocial strategy to treat chronic pain management.

### 7.3 Low Back Pain

MDT has the strongest evidence for the management of low back pain, especially in patients who display directional preference and symptom centralization during symptom assessment. A systematic review by May and Donelson has shown that MDT-based interventions yield comparable or better short-term outcomes than strengthening programs. Failure to centralize is a strong prognostic indicator of higher disability, poor outcomes, and increased psychosocial distress. These findings support the idea that MDT is most effective in treating mechanically driven spinal pain rather than centrally mediated pain [32]. A multitude of clinical guidelines, systematic reviews, and randomized controlled trials have shown that patients who underwent MDT experienced statistically significant reductions in pain and disability at 1 week compared with those who received passive therapies. However, it did not outperform strengthening, flexion exercises, and other active interventions in patients with chronic lower back pain. This displays that the effectiveness of MDT is truly uncertain until it is used as a classification-based approach [33]. While MDT is not a superior solution for all patients with lower back pain, the benefits are most noticeable in specific patient populations. Therefore, MDT is best used as a targeted intervention in a wide rehabilitation framework.

### 7.4 Neck Pain

Similarly to how MDT was applied to lower back pain, it was studied in patients with mechanical neck pain. A review of several randomized clinical trials assessed whether passive cervical spine stretching could reduce pain and disability and improve function in adults with neck pain, with or without radicular symptoms. The review concluded that there was insufficient evidence to confidently prescribe mechanical traction as a stand-alone treatment for neck pain, but some trials displayed positive effects when combined with other interventions [34]. Recent systematic reviews that further examined this topic concluded that, although small, there was a statistically significant improvement in pain of all severities for patients receiving MDT compared with control methods. However, MDT did not improve disability in patients compared with control methods. Additionally, MDT for moderate-to-severe pain was clinically and statistically significant, whereas MDT for mild-to-moderate pain was statistically significant only compared with control methods [35]. The principles of patient education, self-management, and symptom-guided exercise programs remain key to MDT’s role in neck pain, favoring active over passive rehabilitation.

### 7.5 MDT Role in Osteoarthritis and Fibromyalgia

Compared with spinal pain conditions, there is no concrete evidence that MDT addresses the pathophysiology of osteoarthritis, which is characterized by joint degeneration. However, few studies have examined this topic in depth. A randomized controlled trial by Rosedale et al. [36], completed in 2014, examined whether directional-preference exercises are an effective exercise intervention for patients with knee osteoarthritis. Results showed that after 2 weeks and 3 months, patients who underwent MDT had significantly improved pain and physical functional scores compared with controls, but further investigation is needed to verify these results [36]. A novel case report by Khemani et al., published in 2022 [37], described the use of MDT as adjuvant therapy in a 41-year-old female with osteoarthritis. Results showed that MDT reduced knee pain and improved range of motion, muscle strength, and performance post-therapy. MDT was used in conjunction with electrotherapy modalities, soft tissue approaches, and therapeutic training to further benefit the patient [37]. Until further evidence demonstrates that MDT can be used as a primary strategy for treating osteoarthritis, it should be selectively incorporated into multidisciplinary rehabilitation programs.

On the other hand, fibromyalgia is defined by widespread pain, central sensitization, and altered pain processing, with symptoms that tend to be non-mechanical and not influenced by specific movement directions. Therefore, the core principles of MDT of directional preference and symptoms centralization are not appropriate to treat the pathophysiology that characterizes fibromyalgia [38]. However, MDT may have a role in patients with fibromyalgia who have mechanical pain conditions. Patients who present with localized neck or back pain with mechanical features may benefit from an MDT-based assessment and intervention. However, formal studies are needed to research this topic further to determine the exercise dosing and see the symptom response, given the pathophysiology of fibromyalgia.

### 7.6 McKenzie Method Versus Standard Physical Therapy

Evaluation of MDT against standard physical therapy suggests MDT may have an advantage in long-term pain reduction and disability improvement in select patient populations. In a randomized controlled trial by Long et al. [31], patients who performed MDT exercises displayed greater reductions in pain and disability compared with those who performed spinal manipulation and non-specific exercises. MDT exercises were paired with the patient’s directional preference [31]. These findings demonstrate that a mechanically related exercise protocol yields more favorable outcomes than generic physical therapy programs. Additionally, Peterson et al. reported that patients treated with MDT required fewer treatment sessions to return to normal function than those treated with alternative methods, resulting in lower healthcare costs to achieve the same goal [38]. Prolonged treatment for chronic musculoskeletal care takes up essential healthcare resources from other individuals.

### 7.7 McKenzie Method Versus Manual Therapy

Compared with manual therapy, MDT achieves comparable or better long-term functional outcomes, even though many patients are self-managed rather than relying on provider-dependent care. A randomized control trial found that MDT-based interventions created short-term improvements in pain and disability like manual therapy, but MDT maintained these functional improvements over time. This is likely due to MDT’s core principle of independent symptom management [39]. MDT’s focus on repeated movement testing, directional preference, and patient education makes it preferable to passive treatments.

### 7.8 McKenzie Method Versus Exercise-Only Programs

The outcome of MDT compared with exercise-only programs depends on the patient population and the presence of directional preference. A study of patients with low back pain who displayed a directional preference found better outcomes with MDT exercises than with non-specific exercise rehabilitation programs. On the other hand, patients displayed comparable improvements regardless of treatment modality when they had no directional preference [40]. MDT’s ability to classify patients based on mechanical response rather than diagnostic labels makes it an effective treatment modality for identifying and treating patients who will benefit from targeted loading strategies.

### 7.9 Combining MDT With Other Therapeutic Approaches

MDT is most effective when combined with other therapeutic pain modalities that address the wide variety of contributors to chronic pain and disability. Consistent functional participation is needed to see improvements with MDT therapy, and this may not occur unless interventions also target behavioral, occupational, and psychosocial domains. Psychosocial factors that influence chronic pain include fear, maladaptive behaviors, and activity avoidance. On the other hand, an MDT assessment can help distinguish mechanically responsive pain from psychosocially influenced pain. MDT can complement other multidisciplinary therapeutic approaches to improve clinical decision-making [28,29].

Occupational therapy plays a crucial role in translating the symptom improvements observed through MDT into functional improvements. Interventions such as energy conservation strategies, activity pacing, and task simplification and prioritization help limit symptom aggravation [41]. Functional emphasis is important in chronic pain populations, since improvements in pain or movement capacity do not necessarily lead to better quality of life [42]. Occupational therapy is a first-line non-pharmacological method part of a multidisciplinary model that improves self-efficacy and long-term self-management through sustainable daily routines and reduces daily limitations. It leads to reduced disability in chronic pain populations affected by musculoskeletal and rheumatological conditions [41].

Psychological interventions further improve the efficacy of MDT by addressing pain-related cognitions and behaviors, including fear avoidance, catastrophizing, and movement-related anxiety. Catastrophizing leads to higher depression and negative mood and disrupts interpersonal functions, leading to greater disability. A common method is cognitive-behavioral therapy, which aims to reduce catastrophizing and improve coping through cognitive restructuring. Less catastrophizing is correlated with 6-month and 12-month enhancements in disability, pain intensity, and depression. Early screening to identify high catastrophizers offers insight into which risk markers to prioritize with psychological support [43].

Pharmacological management serves as an adjunct to a multidisciplinary model to control symptoms while patients undergo an active rehabilitation program. These treatments display a limited long-term effectiveness, and reliance on certain pain medications carries a variety of risks without improving overall function. Various medications include SNRIs, NSAIDs, TCAs, and gabapentoids, which help lower pain intensity and allow patients to engage in rehabilitation regimens such as MDT [21,22]. Current guidelines recommend not using pharmacological therapy alone, as it does not lead to long-term functional improvement. It is most effective when combined with both a rehabilitation program and behavioral interventions, restoring function and improving quality of life [23,24].

**Figure 2:**
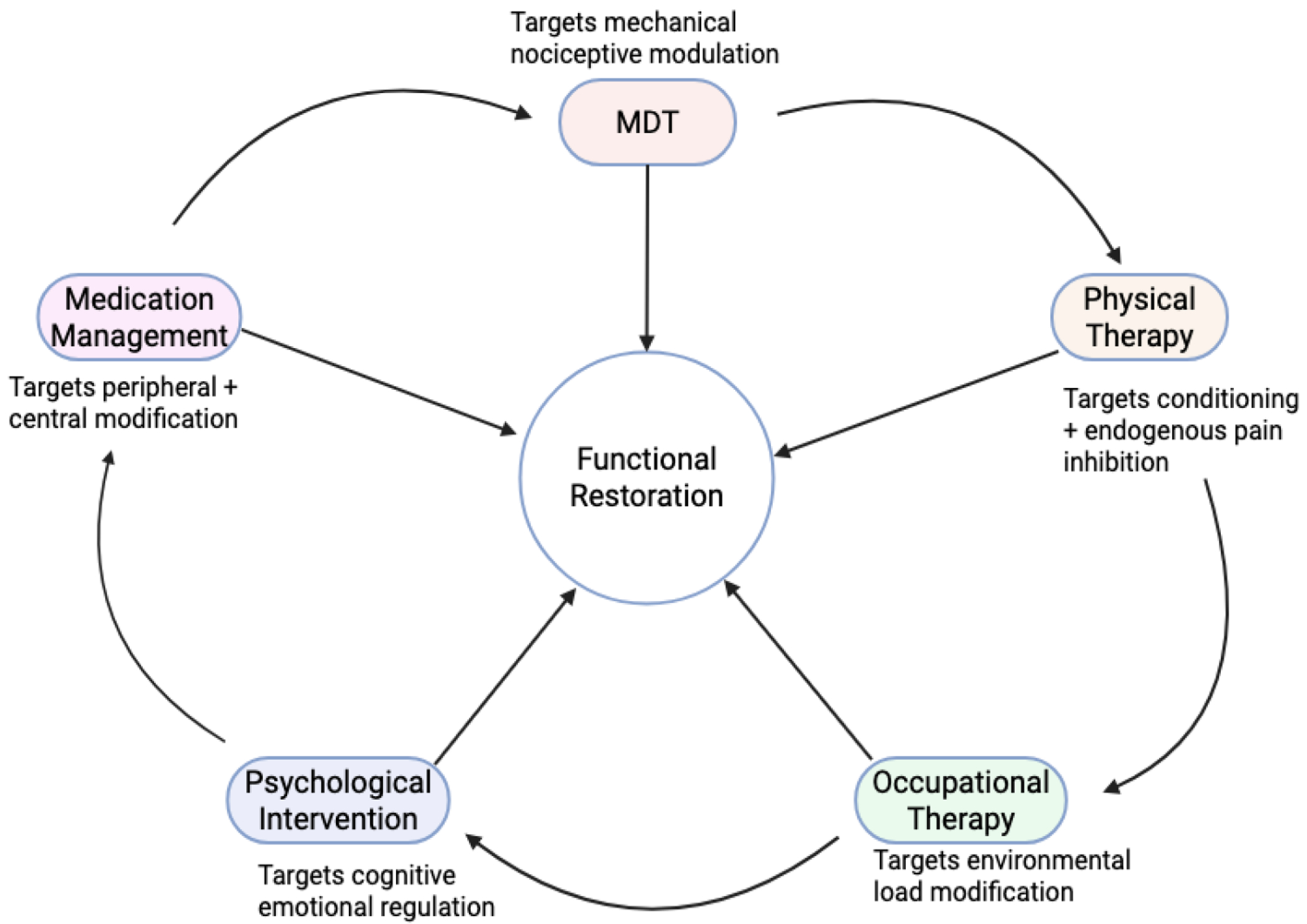
Multidisciplinary Integration Wheel: All these factors work together in the overall functional restoration of an individual.

Lastly, progressive strength-training programs are required to sustain and further the gains achieved through MDT. MDT limits pain and improves range of movement in the short- to medium-term by focusing on the mechanical contributors to pain (Figure 2). Long-term exercise will improve one’s functional capacity and physical function over time [14,15]. Multidisciplinary rehabilitation programs that incorporate MDT, behavioral strategies, and physical therapy to treat chronic pain conditions characterized by pain and disability demonstrate favorable long-term outcomes compared with unimodal interventions [27,28] (Figure 2).

## 8. Limitations and Areas for Future Research

Despite extensive research on MDT’s role in spinal pain caused by mechanical mechanisms, the use of MDT in centrally mediated conditions such as fibromyalgia and osteoarthritis remains poorly studied. Current studies do not support MDT as the primary therapeutic option for these conditions, with most research not accounting for patients with widespread pain or inflammatory diseases. Therefore, the data on the effectiveness of MDT cannot be extrapolated to this patient population, allowing it only to be considered an adjunctive therapy when a patient also presents with mechanical pain.

Additionally, upcoming studies should evaluate MDT within a multidisciplinary framework rather than as a unimodal intervention. Randomized controlled trials investigating MDT integrated with occupational therapy, physical therapy, psychological interventions, and medication management are needed to understand MDT’s long-term effects and enable its more widespread use. New forms of health care delivery, such as tele-rehabilitation and technology-assisted self-management, can expand MDT’s use beyond the conventional clinical setting. Lastly, addressing gaps in limited long-term follow-up and inadequate integration of behavioral outcomes can play a crucial role in highlighting MDT’s effect within a biopsychosocial model of chronic pain management.

## 9. Conclusion

MDT is an effective therapeutic model for managing mechanical spine pain in patients with movement-dependent symptoms, directional preference, and centralization. MDT used in these populations has led to reduced pain and the use of passive treatment modalities, leading to self-management and better functional outcomes. This is the key reason to integrate MDT into modern rehabilitation methods [31–33]. However, MDT has limited effects in patients with diseases characterized by systemic and centrally mediated factors, seen in fibromyalgia and osteoarthritis. These diseases are described by inflammation, structural joint pathology, and altered central pain processing. [21,22,24]. MDT may still be used in these patients with coexisting movement impairments. Long-term solutions to treat chronic musculoskeletal pain are effective when comprised of a multidisciplinary model consisting of a biopsychosocial approach, including physical therapy, occupational therapy, medication management, and psychological interventions [27,28]. Therefore, MDT is best used as a mechanism-specific, patient-centered tool within a multimodal rehabilitation program, with the goal of restoring function, self-efficacy, and long-term participation.

## Key points

- Chronic musculoskeletal pain emerges from biological, psychological, and social interactions
- Osteoarthritis is degenerative and inflammatory, while fibromyalgia involves central sensitization.
- Multidisciplinary rehabilitation leads to better long-term outcomes in comparison to a singular model of care
- Physical therapy improves strength, conditioning, and movement tolerance while occupational therapy increases adaptation to one’s environment.
- Psychological intervention lowers fear-avoidance and catastrophizing, while pharmacological therapies help control symptoms, but have limited long-term functional restoration
- MDT classifies patients in derangement, dysfunction, and postural syndromes
- MDT is seen to have the strongest effects in mechanically mediated low back pain
- Centralization and directional preference are key factors that predict the success of MDT
- Evidence supporting MDT in osteoarthritis and fibromyalgia is limited and remains an adjunctive technique

## References

[1] Verhaar JB. Degenerative and Inflammatory Joint Diseases. In: Verhaar JAN, Kjærsgaard-Andersen P, Limb D, The EFORT White Book: Orthopaedics and Traumatology in Europe. Lowestoft (UK): Dennis Barber Ltd (2021). 36327376

[2] Aabedi A, Fraix MP, Agrawal DK. Surgical Interventions in Severe Osteoarthritis: Pros and Cons. J Orthop Sports Med 7 (2025): 169–178. 40406238 10.26502/josm.511500192 PMC12097792

[3] Rajalekshmi R, Agrawal DK. Advancing Osteoarthritis Research: Insights from Rodent Models and Emerging Trends. J Orthop Sports Med 7 (2025): 110–128. 40264810 10.26502/josm.511500187 PMC12014194

[4] Enni JBA, Agrawal DK. Application of Artificial Intelligence and Its Subsets in Various Stages of Knee Arthroplasty from Pre-op to Post-op: An Overview. J Orthop Sports Med 7 (2025): 96–102. 40190766 10.26502/josm.511500185 PMC11970954

[5] Abdou A, Kades S, Masri-Zada T, Lumbar Spinal Stenosis: Pathophysiology, Biomechanics, and Innovations in Diagnosis and Management. J Spine Res Surg 7 (2025): 1–17. 40083985 10.26502/fjsrs0082 PMC11906179

[6] Rai V, Dilisio MF, Agrawal DK. Counteractive Effects of IL-33 and IL-37 on Inflammation in Osteoarthritis. Int J Environ Res Public Health 19 (2022): 5690. 35565085 10.3390/ijerph19095690 PMC9100324

[7] Garfinkel RJ, Dilisio MF, Agrawal DK. Vitamin D and Its Effects on Articular Cartilage and Osteoarthritis. Orthop J Sports Med 5 (2017): 2325967117711376. 10.1177/2325967117711376 PMC5480771 28680892

[8] Rosenberg JH, Rai V, Dilisio MF, Damage-Associated Molecular Patterns in the Pathogenesis of Osteoarthritis: Potentially Novel Therapeutic Targets. Mol Cell Biochem 434 (2017): 171–179. 28474284 10.1007/s11010-017-3047-4 PMC5671379

[9] Rosenberg JH, Rai V, Dilisio MF, Increased Expression of Damage-Associated Molecular Patterns (DAMPs) in Osteoarthritis of Human Knee Joint Compared to Hip Joint. Mol Cell Biochem 436 (2017): 59–69. 28573383 10.1007/s11010-017-3078-x

[10] Rai V, Dietz NE, Dilisio MF, Vitamin D Attenuates Inflammation, Fatty Infiltration, and Cartilage Loss in the Knee of Hyperlipidemic Microswine. Arthritis Res Ther 18 (2016): 203. 27624724 10.1186/s13075-016-1099-6 PMC5022245

[11] Sen R, Hurley JA. Osteoarthritis. StatPearls. Treasure Island (FL): StatPearls Publishing (2025).

[12] Bhargava J, Goldin J. Fibromyalgia. StatPearls. Treasure Island (FL): StatPearls Publishing (2025).

[13] Institute for Quality and Efficiency in Health Care. In Brief: Physical Therapy. Cologne, Germany: IQWiG (2024).

[14] Busch AJ, Webber SC, Brachaniec M, Exercise Therapy for Fibromyalgia. Curr Pain Headache Rep 15 (2011): 358–367. 21725900 10.1007/s11916-011-0214-2 PMC3165132

[15] Fransen M, McConnell S, Harmer AR, Exercise for Osteoarthritis of the Knee: A Cochrane Systematic Review. Br J Sports Med 49 (2015): 1554–1557. 26405113 10.1136/bjsports-2015-095424

[16] Deyle GD, Henderson NE, Matekel RL, Effectiveness of Manual Physical Therapy and Exercise in Osteoarthritis of the Knee: A Randomized Controlled Trial. Ann Intern Med 132 (2000): 173–181. 10651597 10.7326/0003-4819-132-3-200002010-00002

[17] Institute for Quality and Efficiency in Health Care. In Brief: What Is Occupational Therapy? Cologne, Germany: IQWiG (2024).

[18] Bolt M, Ikking T, Baaijen R, Occupational Therapy and Primary Care. Prim Health Care Res Dev 20 (2019): e27. 32799974 10.1017/S1463423618000452 PMC6476805

[19] Clark BM. Rheumatology: Physical and Occupational Therapy in the Management of Arthritis. CMAJ 163 (2000): 999–1005. 11068573 PMC80550

[20] Jaicks CCD. Evaluating the Benefits of Occupational Therapy in Children with Autism Spectrum Disorder Using the Autism Behavior Checklist. Cureus 16 (2024): e64012. 39109123 10.7759/cureus.64012 PMC11302171

[21] Ablin J, Fitzcharles MA, Buskila D, Treatment of fibromyalgia syndrome: recommendations of recent evidence-based interdisciplinary guidelines with special emphasis on complementary and alternative therapies. Evidence-Based Complementary and Alternative Medicine 2013 (2013): 485272. 10.1155/2013/485272 PMC3856149 24348701

[22] Clauw DJ. Fibromyalgia: a clinical review. JAMA 311 (2014): 1547–1555. 24737367 10.1001/jama.2014.3266

[23] Smolen JS, Landewé RBM, Bergstra SA, EULAR recommendations for the management of rheumatoid arthritis with synthetic and biological disease-modifying antirheumatic drugs: 2022 update. Annals of the Rheumatic Diseases 82 (2023): 3–18. 36357155 10.1136/ard-2022-223356

[24] Hunter DJ, Bierma-Zeinstra S. Osteoarthritis. The Lancet 393 (2019): 1745–1759. 10.1016/S0140-6736(19)30417-9 31034380

[25] Bernardy K, Füber N, Köllner V, Efficacy of cognitive-behavioral therapies in fibromyalgia syndrome: a systematic review and meta-analysis of randomized controlled trials. Journal of Rheumatology 37 (2010): 1991–2005. 20682676 10.3899/jrheum.100104

[26] Garland EL, Hanley AW, Nakamura Y, Mindfulness-Oriented Recovery Enhancement vs Supportive Group Therapy for co-occurring opioid misuse and chronic pain in primary care: a randomized clinical trial. JAMA Internal Medicine 182 (2022): 407–417. 35226053 10.1001/jamainternmed.2022.0033 PMC8886485

[27] Gatchel RJ, Peng YB, Peters ML, The biopsychosocial approach to chronic pain: scientific advances and future directions. Psychological Bulletin 133 (2007): 581–624. 17592957 10.1037/0033-2909.133.4.581

[28] Kamper SJ, Apeldoorn AT, Chiarotto A, Multidisciplinary biopsychosocial rehabilitation for chronic low back pain: Cochrane systematic review and meta-analysis. BMJ 350 (2015): h444. 25694111 10.1136/bmj.h444 PMC4353283

[29] Lam OT, Strenger DM, Chan-Fee M, Effectiveness of the McKenzie Method of Mechanical Diagnosis and Therapy for treating low back pain: literature review with meta-analysis. Journal of Orthopaedic and Sports Physical Therapy 48 (2018): 476–490. 29602304 10.2519/jospt.2018.7562

[30] Mann SJ, Stretanski MF, Singh P. McKenzie Back Exercises. StatPearls Publishing (2025).

[31] Long A, Donelson R, Fung T. Does it matter which exercise? A randomized controlled trial of exercise for low back pain. Spine 29 (2004): 2593–2602. 15564907 10.1097/01.brs.0000146464.23007.2a

[32] May S, Donelson R. Evidence-informed management of chronic low back pain with the McKenzie method. Spine Journal 8 (2008): 134–141. 10.1016/j.spinee.2007.10.017 18164461

[33] Machado LA, de Souza MV, Ferreira PH, The McKenzie method for low back pain: a systematic review of the literature with a meta-analysis approach. Spine 31 (2006): E254–E262. 16641766 10.1097/01.brs.0000214884.18502.93

[34] Graham N, Gross A, Goldsmith CH, Mechanical traction for neck pain with or without radiculopathy. Cochrane Database of Systematic Reviews (2008): CD006408. 18646151 10.1002/14651858.CD006408.pub2 PMC13429286

[35] Baumann AN, Orellana K, Landis L, The McKenzie Method is an effective rehabilitation paradigm for treating adults with moderate-to-severe neck pain: a systematic review with meta-analysis. Cureus 15 (2023): e39218. 37337494 10.7759/cureus.39218 PMC10276901

[36] Rosedale R, Rastogi R, May S, Efficacy of exercise intervention as determined by the McKenzie System of Mechanical Diagnosis and Therapy for knee osteoarthritis: a randomized controlled trial. Journal of Orthopaedic and Sports Physical Therapy 44 (2014): 173–A6. 24450370 10.2519/jospt.2014.4791

[37] Khemani S, Shah S, Mhase S, Pragmatic effect of lower limb McKenzie in grade one osteoarthritis: a novel case report. Cureus 14 (2022): e29945. 36348925 10.7759/cureus.29945 PMC9635331

[38] Petersen T, Kryger P, Ekdahl C, The effect of McKenzie therapy as compared with that of intensive strengthening training for the treatment of patients with subacute or chronic low back pain: a randomized controlled trial. Spine 27 (2002): 1702–1709. 12195058 10.1097/00007632-200208150-00004

[39] Halliday MH, Pappas E, Hancock MJ, A randomized controlled trial comparing the McKenzie Method to motor control exercises in people with chronic low back pain and a directional preference. Journal of Orthopaedic and Sports Physical Therapy 46 (2016): 514–522. 27170524 10.2519/jospt.2016.6379

[40] Werneke MW, Edmond S, Young M, Directional preference and functional outcomes among subjects classified at high psychosocial risk using STarT. Physiotherapy Research International 23 (2018): e1711. 29536595 10.1002/pri.1711

[41] Smolen JS, Landewé RBM, Bijlsma JWJ, EULAR recommendations for the management of rheumatoid arthritis with synthetic and biological disease-modifying antirheumatic drugs: 2019 update. Annals of the Rheumatic Diseases 79 (2020): 685–699. 31969328 10.1136/annrheumdis-2019-216655

[42] Waddell G, Burton AK. Concepts of rehabilitation for the management of low back pain. Best Practice and Research Clinical Rheumatology 19 (2005): 655–670. 15949782 10.1016/j.berh.2005.03.008

[43] Quartana PJ, Campbell CM, Edwards RR. Pain catastrophizing: a critical review. Expert Review of Neurotherapeutics 9 (2009): 745–758. 19402782 10.1586/ERN.09.34 PMC2696024

